# Auricular reconstruction after Mohs excision utilizing combination of pre-auricular transposition and chondrocutaneous advancement flaps

**DOI:** 10.1080/23320885.2022.2026776

**Published:** 2022-01-17

**Authors:** Annet S. Kuruvilla, Jared M. Gopman, Peter W. Henderson

**Affiliations:** Division of Plastic and Reconstructive Surgery, Icahn School of Medicine at Mount Sinai, New York, NY, USA

**Keywords:** Auricular reconstruction, coronavirus, flaps, Mohs procedure

## Abstract

Auricular reconstruction can pose a challenge for any well-trained plastic surgeon, especially with the COVID-19 pandemic and pressure to decrease stages and office visits. The case report involves a single-stage reconstruction of the auricular upper-third in an elderly male using a unique combination of pre-auricular fasciocutaneous transposition and chondrocutaneous advancement flaps.

## Introduction

The ear is prone to the development of skin cancer due to prolonged sun exposure, and reconstruction of resulting acquired defects after surgical excision can be challenging. Despite the abundance of literature that exists detailing ear reconstruction [1,2,3–19], no single surgical plan befits all reconstructive needs and individualized treatment plans are necessary. Single-stage auricular reconstruction can be particularly challenging due to the distinct anatomy of the ear and the need to prioritize both function and aesthetic [20]. Single-stage reconstructive sequences can be favorable in the elderly population and for those with comorbidities. Furthermore, the COVID-19 pandemic introduces unique considerations, such as the goal of minimizing the number of stages and healthcare visits, as well as maintaining the ability to wear masks that wrap around the ear.

The case reported herein utilizes a novel combination of local flaps to achieve the successful single-stage reconstruction of a large defect of the auricular upper-third in an elderly patient during the COVID-19 pandemic.

## Case report

**Figure 1.**
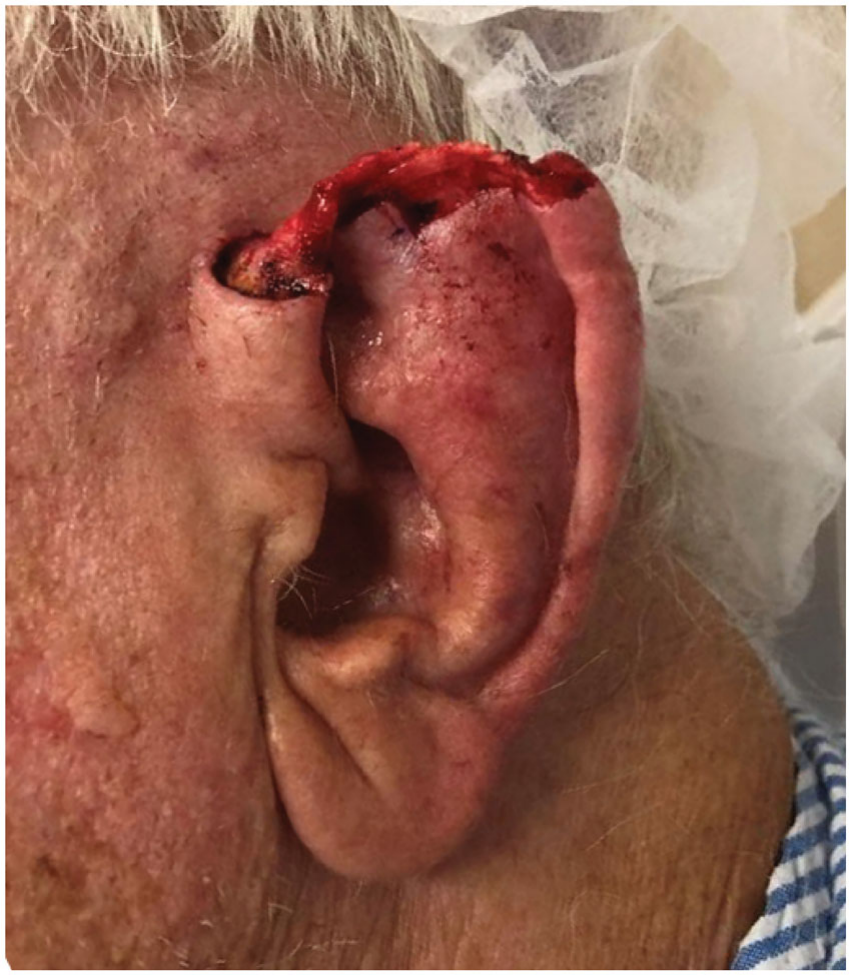
Patient initially presented with 4.2 × 2.5 cm (10.5 cm^2^) auricular skin defect of the superior helix and superior crus of the scapha that extends posteriorly into the superior retro-auricular sulcus after Mohs micrographic surgery excision of a basal cell carcinoma.

A 73-year-old man with multiple medical comorbidities presented in September 2020 for immediate left ear reconstruction due to a 4.2 × 2.5 cm (10.5 cm^2^) skin defect after Mohs micrographic surgery excision of basal cell carcinoma. The full-thickness skin defect of the superior helix and superior crus of the helix extended posteriorly into the superior retro-auricular sulcus (Figure 1). The goals of reconstruction included the improved structural shape of the ear (in isolation, not necessarily with the goal of achieving symmetry with the right ear), recruitment of skin to the anterior and posterior portions of the superior ear without distorting the donor site, maintaining the ability to wear his prescription glasses, a reconstruction that does not inhibit continued mask wearing for SARS-COV-2 protection, and an ambulatory operation with minimal anesthesia risk that could be performed as a single-stage procedure in order to decrease the number of healthcare visits during the COVID-19 pandemic.

**Figure 2.**
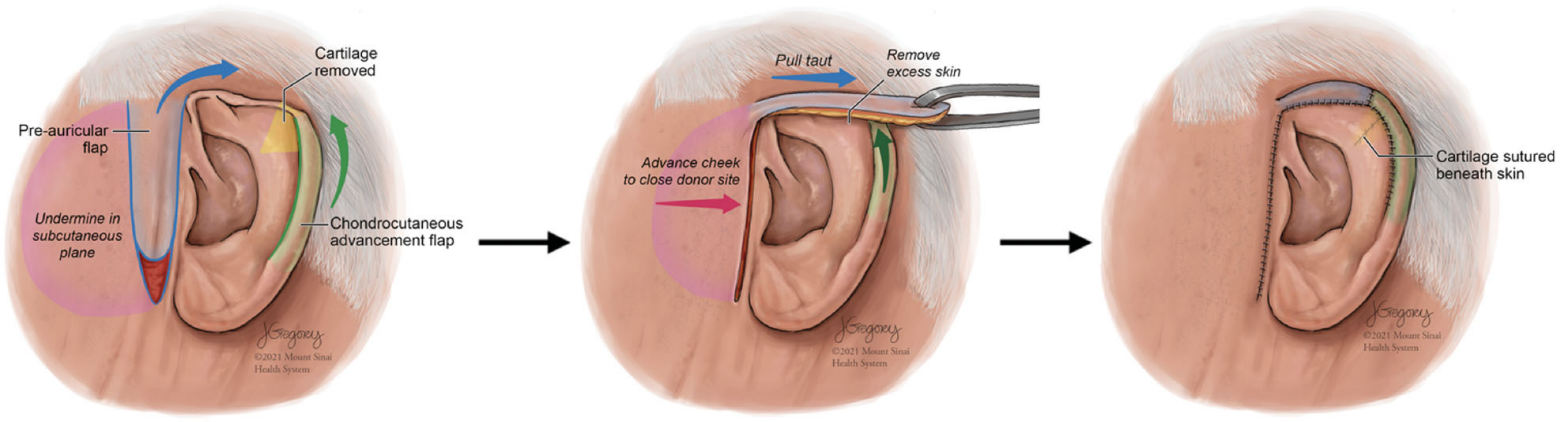
Diagram showing the repair procedure consisting of a unilateral chondrocutaneous advancement (Antia-Buch) flap to improve the shape of the ear and a pre-auricular fasciocutaneous transposition flap to provide skin coverage.

A unique combination procedure was designed that consisted of a unilateral chondrocutaneous advancement (Antia-Buch) flap (to improve the shape of the ear) and a pre-auricular fasciocutaneous transposition flap (to provide skin coverage) (Figure 2). The Antia-Buch flap was designed along the lateral helical rim, the posterior skin was kept intact as the vascular support to the helical rim cartilage, the cartilage was advanced, and a small amount of cartilage was debrided and removed as a wedge extending into the scapha to allow for appropriate closure without disruption of normal contour. Separately, a superiorly-based pre-auricular fasciocutaneous transposition flap was raised superficially to the superficial musculoaponeurotic system (SMAS)/parotid fascia. The flap was rotated approximately 90° and draped over the helical root, and inset with 5-0 nylon sutures to the leading edge of the helical rim chondrocutaneous advancement flap.

**Figure 3.**
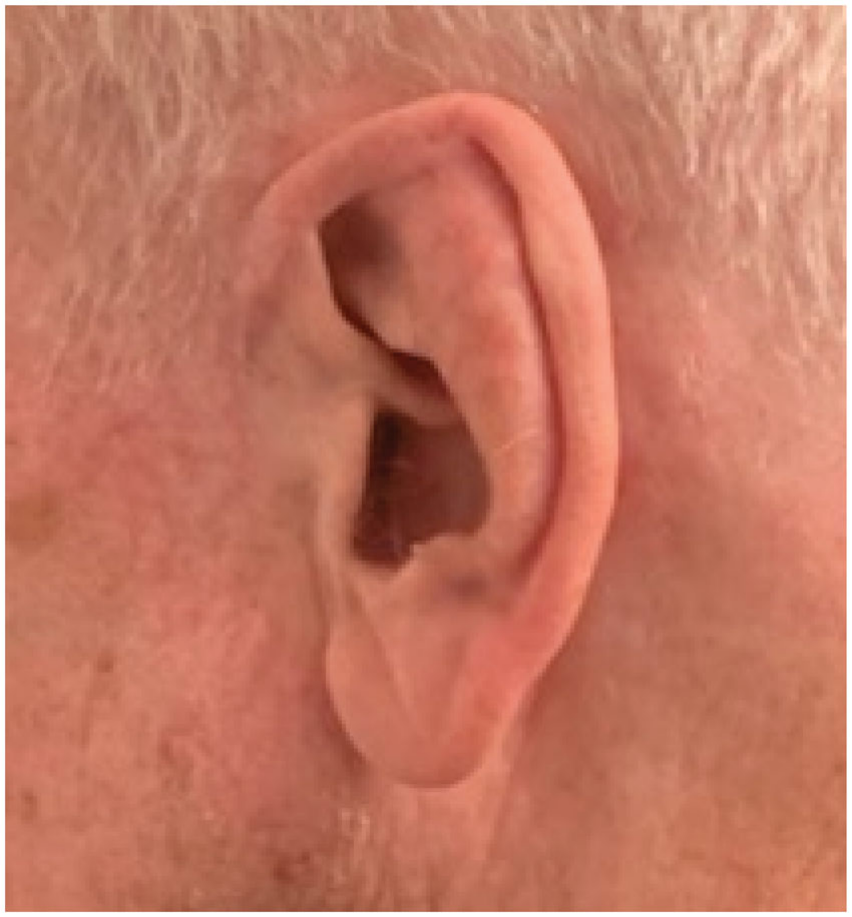
Postoperative photo (12 months) results.

Twelve months postoperatively, the surgical sites healed uneventfully, he was pleased with the results, and he had been able to wear his mask at all times in the postoperative period (Figure 3).

## Discussion

Small defects of the helical rim in the upper third can be managed by the Antia-Buch chondrocutaneous helical advancement flap, and this can usually be completed in a single stage [13]. The creation of this flap involves an anterior incision along the helical to separate the helix and the scapha. A superficial dissection to the depth of the perichondrium is made in the posterior auricle in order to create the flaps that will converge at the wedge cut in the antihelix [21]. First described for helical rim defects up to 20 mm in size, larger defects have been repaired using modifications of this flap such as V-Y advancement of the helical root [7,14]. Larger defects repaired with an Antia-Buch flap can lead to a cup ear deformity, resulting in a superior helical rim that is folded over inferiorly. Modifications to the original methodology include the utilization of wedge cuts in the antihelix and chondra in order to avoid undesirable outcomes [21]. When larger defects extend beyond the helical rim of the upper third into the scapha and antihelix, single-stage pedicled chondrocutaneous transposition flaps based on the root of the helix or the caudal part of the helix have been described by Davis [15] and Orticochea [16], respectively.

The superiorly-based pre-auricular fasciocutaneous transposition flap, a random-pattern flap designed to hide the donor site scar in the pre-auricular sulcus, is recommended to not exceed a 3:1 length-to-width ratio to prevent insufficient vascularity at the distal tip. This is often not a limiting factor, however, as there typically exists an abundance of donor site tissue especially in elderly patients secondary to facial aging, and the donor site can be closed easily via cheek advancement. This flap can resurface nearly the entire superior helical rim including the uppermost scapha anteriorly and the superior retro-auricular sulcus posteriorly. However, as this flap lacks any cartilaginous framework, the superior helical rim would likely lose its normal convexity without either an underlying cartilage graft or by suspension at the flap tip during inset. This underscores the need for the helical chondrocutaneous advancement flap, which is inset superiorly to the pre-auricular flap, which itself is inset on gentle stretch to hold somewhat taut and prevent a disfiguring concavity of the superior helix. Another drawback of the pre-auricular fasciocutaneous transposition flap, especially in male patients, is the possible placement of hair-bearing skin on the superior helix as in our patient. Patients must be counseled of this possibility prior to surgery, and can self-remove the hair or elect to undergo electrolysis once the flap has sufficiently healed.

This is not the first report on the use of multiple local flaps for reconstruction of a large upper-third auricular defect. Yotsuynagi et al. described using a conchal chondrocutaneous transposition flap as described by Davis [15] combined with a transposition flap of upper post-auricular skin to cover the superior retro-auricular sulcus defect [19]. Unlike the techniques described by Yotsuynagi et al., combining a pre-auricular fasciocutaneous advancement flap with a chondrocutaneous helical rim advancement flap precludes the need for post-auricular tissue for reconstruction, preventing scar burden at the site of elastic compression from mask usage. Whereas the post-auricular blood supply was left intact as originally described by Antia and Buch, modifications of the inferior chondrocutaneous helical advancement flap include incision of postauricular skin and advancement based on a long, narrow pedicle [7].

This innovative methodology of combining the laterally-based helical chondrocutaneous advancement flap and a medially-based pre-auricular fasciocutaneous transposition flap in a single stage has shown to be a viable solution with an aesthetically pleasing and functional outcome. Local flaps from pre- or post-auricular skin are ideal for vascularized soft tissue coverage in denuded cartilage. However, these may have some degree of color mismatch, require multiple stages, or place new thicker skin which can obscure native cartilaginous contour [1,8,9]. The limitations of this study include the focus upon exploring a single stage reconstructive method as opposed to other methodologies that may be multistage, but offer the potential to be more efficacious. The evaluation criteria for this procedure was maintaining the original integrity and shape of the ear, negligible distortion of the donor site, and maintaining the ability to wear his prescription glasses and mask all while achieving a single-stage flap as an ambulatory procedure.

In the age of the COVID-19 pandemic, where mask wearing has become commonplace and most masks involve wrapping an elastic band around the post-auricular sulcus, the ability to hold the mask in place with an intact upper auricular framework is even more necessary for public health measures. Other day to day necessities requiring a sound upper auricle, particularly in the geriatric population, include the use of hearing aids and glasses. The constant use of a mask throughout one’s day, especially those in the field of health care, can lead to constant friction and irritation of the skin in the post-auricular area. In some cases, this has even resulted in a pressure injury [22]. Therefore, having an option of having a single-stage auricular reconstruction can prove to be beneficial in these times in order to ensure efficient healing with minimal complications that can be exacerbated with the daily use of a mask. Over time, the repair is predetermined to hold its structure and have minimal foreseeable complications pending no skin cancer recurrence and no trauma.

The combination of a laterally-based helical chondrocutaneous advancement flap and a medially-based pre-auricular fasciocutaneous transposition flap can be successfully performed in a single stage to reconstruct composite defects of the superior ear. Furthermore, this approach has particular benefits in the coronavirus period and should be considered as a valuable option for superior helical reconstruction.
